# Cultural Diversity, Economic Development and Societal Instability

**DOI:** 10.1371/journal.pone.0000929

**Published:** 2007-09-26

**Authors:** Daniel Nettle, James B. Grace, Marc Choisy, Howard V. Cornell, Jean-François Guégan, Michael E. Hochberg

**Affiliations:** 1 Centre for Behaviour and Evolution, Newcastle University, Newcastle, United Kingdom; 2 U.S. Geological Survey, Lafayette, Louisiana, United States of America; 3 Institute of Ecology, University of Georgia, Athens, Georgia, United States of America; 4 Génétique et Evolution des Maladies Infectieuses, UMR 2724 IRD-CNRS, Montpellier, France; 5 Department of Environmental Science and Policy, University of California at Davis, Davis, California, United States of America; 6 Institut des Sciences de l'Evolution, UMR 5554,Université Montpellier II, Montpellier, France; 7 National Center for Ecological Analysis and Synthesis, Santa Barbara, California, United States of America; 8 Santa Fe Institute, Santa Fe, New Mexico, United States of America; Georgia State University, United States of America

## Abstract

**Background:**

Social scientists have suggested that cultural diversity in a nation leads to societal instability. However, societal instability may be affected not only by within-nation or α diversity, but also diversity between a nation and its neighbours or β diversity. It is also necessary to distinguish different domains of diversity, namely linguistic, ethnic and religious, and to distinguish between the direct effects of diversity on societal instability, and effects that are mediated by economic conditions.

**Methodology/Principal Findings:**

We assembled a large cross-national dataset with information on α and β cultural diversity, economic conditions, and indices of societal instability. Structural equation modeling was used to evaluate the direct and indirect effects of cultural diversity on economics and societal stability. Results show that different types and domains of diversity have interacting effects. As previously documented, linguistic α diversity has a negative effect on economic performance, and we show that it is largely through this economic mechanism that it affects societal instability. For β diversity, the higher the linguistic diversity among nations in a region, the less stable the nation. But, religious β diversity has the opposite effect, reducing instability, particularly in the presence of high linguistic diversity.

**Conclusions:**

Within-nation linguistic diversity is associated with reduced economic performance, which, in turn, increases societal instability. Nations which differ linguistically from their neighbors are also less stable. However, religious diversity between neighboring nations has the opposite effect, decreasing societal instability.

## Introduction

Ethnic divisions are often invoked to explain civil strife and conflict, but what evidence implicates cultural diversity as a causal factor in such strife? Social scientists have often argued that diversity within a nation might have negative effects on societal outcomes. Ethnic cleavages within a nation can create barriers to communication and exchange, factions and rivalries, and internal conflict [1], [2]. On the other hand, theory predicts that social homogeneity will encourage the formation of social capital or trust [3].

There have been surprisingly few direct empirical studies of how cultural diversity affects social instability. The focus has instead been on the relationship between cultural diversity and economic performance across nations. Generally, the relationship is weakly negative, whether economic performance is measured as national wealth (GNP or GDP; [4], [5]), productivity [6] or economic growth [1], [7] (though see [8]).

In this paper, we examine the relationship between cultural diversity and societal instability using a large cross-national data set. We used revolutions, coups, civil wars, and other types of serious political strife as indices of societal instability. Any effect of cultural diversity on societal instability could operate indirectly *via* its previously-documented effects on economic performance. Alternatively, cultural diversity could have a direct effect on societal instability, un-mediated by economic factors.

In addition to taking societal instability, rather than economic performance, as the outcome variable, our study extends previous work in three important ways. First, cultural diversity has variously been defined linguistically, ethnically, and in terms of religious affiliation. A recent study comparing these types of diversity [7] concludes that religious diversity has very different effects from those of ethnic or linguistic diversity. Thus we explore the differential effects of all three types of diversity simultaneously.

Second, we employ more sophisticated measures of diversity than previous studies. In particular, we draw out the distinction from ecology between internal, or α, diversity, and external, or β, diversity. Alpha diversity, for which there are several possible metrics (see Methods) is related to the probability that any two citizens within a nation come from the same cultural group. Beta diversity, on the other hand, is related to the probability that a given cultural group found in the nation is also found in the neighboring nations. Thus, α is high when there are many different groups within a nation, and β is high when the groups in a nation are very different from those in the surrounding nations.

Finally, we employ more sophisticated analytic tools than previous studies, which have generally used simple correlation or regression methods. These permit the identification of additive linear effects of multiple independent variables on an outcome, but cannot identify complex causal paths. In particular, they cannot distinguish whether predictor variables have direct effects on outcomes or indirect effects via intermediary variables, which is precisely the question of interest here. We therefore use structural equation modeling (SEM) to examine different causal possibilities. SEM is a multiequational modeling system suitable for asking complex questions about the responses of systems to interconnected sets of explanatory factors [9], [10]. Using SEM we probed the contributions of multiple cultural diversity measures to international variations in economics and societal instability. We evaluated the direct and indirectly-mediated effects of linguistic, religious, and ethnic diversity (both within-nations and across-nations) on indicators of societal instability while controlling for the correlated effects of population size and number of borders.

**Figure 1:**
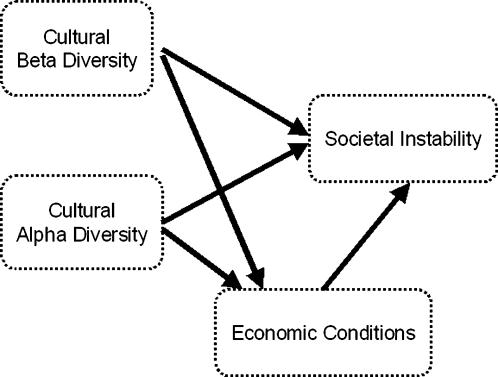
The conceptual structure of the a priori models examined. Not shown are the controlled effects of population size and the number of borders a nation possesses.

To summarize, there are four groups of variables which are of interest: cultural α diversities, cultural β diversities, economic measures, and proxies of societal instability. We wish to examine the direct and indirect (through economic conditions) effects of α and β cultural diversity on societal instability. The conceptual model underlying our analysis is thus that shown in figure 1.

## Methods

### Data set

A cross-national data set was assembled for 212 nations from three sources [11]–[13]. Not all nations have measured values for some of the variables. (Methods for handling missing data are described in Statistical Analyses, below). Our data include several proxies for national wealth, cultural diversity, social instability, and basic demographic and geographic parameters. Proxies for national wealth were per capita GDP, and Gini, the coefficient of income inequality. Some previous studies have used the rate of GDP growth over a specified period rather than GDP itself as a measure of economic performance (e.g. [8]), but we believe that as a measure of very long-term economic success, GDP itself is more informative (see [5], [14]). Diversities were calculated for language, religious and ethnic groups (see Diversity Indices, below). Proxies for societal instability were an index of overall political instability (PInstab), and an index of the occurrence of revolutions and coups d'états (RevCoup) drawn from [11].

### Diversity indices

We separately consider diversity indices for language, ethnicity, or religion and examine their interrelationships as part of the analysis. Data on religious and ethnic diversity are from [13], and for language diversity, from [12].

For the purposes of this study, α diversity is the cultural diversity within a nation. We use a common index of species diversity from the ecology literature, Simpson's *D*, which takes into account not only the number of groups in the assemblage but also their relative abundance. Simpson's *D* is calculated by first determining the proportion *p_i_* of the total number of individuals in the assemblage represented by each species *i*. These values are next squared and summed over all species, *S* to obtain 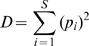. The quantity *D* is simply the probability that two individuals chosen at random from the assemblage represent the same group. As diversity increases, *D* decreases, so the index is usually presented as *1-D* [15] which we use in our study. We also calculated an alternative index of α diversity, Shannon's *H*. Shannon's *H* correlates with Simpson's *D* at around *r* = 0.99, so we consider it no further here.

For β diversity, we calculated Jaccard's coefficient of similarity, defined as 
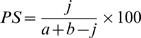
, where *PS* is the percentage similarity between assemblages, *j* is the number of species shared by the assemblages, *a* is the number of species in the first assemblage and *b* is the number in the second assemblage [15]. The index ranges from 0% when no species are shared to 100% when the compositions of the assemblages are identical. As beta diversity increases, *PS* decreases, thus we employ 1-*PS* in the analyses. We calculated mean Jaccard coefficients for each nation and all of its neighbours for religious, language, and ethnic groups. Note that β diversity for islands is undefined without additional assumptions so islands had no β diversity scores in our dataset. We also calculated an alternative index of β diversity, Sorenson's coefficient [15], but as it correlated with Jaccard's at around *r* = 0.99, we consider it no further.

### Statistical analyses

We used the *Structural Equation Modeling* (SEM) software package Amos 7.0 [16] to assess the influence of cultural diversity on social stability. Our a priori model followed the general specification shown in Figure 1. Missing values were handled using the full information method described in [17], which allows all available data to be incorporated in the estimation process while taking no active steps toward estimating missing values. In our specification of the a priori model, we allowed for nonlinear path relations using the composite modeling process described in [18]. In addition, we allowed for evaluation of the interactions between diversity components. Model evaluations were based on *Χ^2^* statistics that measure the discrepancy between observed and model-implied covariance matrices. In addition to initial assessments of overall model fit, the significances of individual paths were assessed using single-degree-of-freedom *Χ^2^* tests. Finally, we considered whether there was any indication that political instability actually drives the variations among nations in GDP (rather than vice versa) by evaluating a model that included such a feedback.

## Results

**Figure 2:**
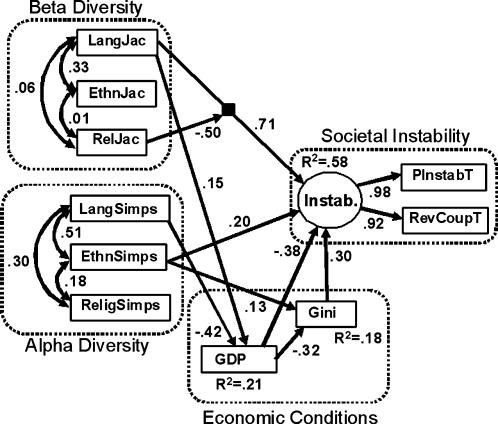
SEM results, indicating the relationships between variables and constructs (dotted boxes). Model *Χ^2^* = 31.7 with 34 df, p = 0.581 (indicating close fit between model and data). Coefficients shown are standardized values.

The structural equation model results from our analyses are shown in Figure 2 (see also Table 1). For direct paths, only statistically significant pathways are displayed (intercorrelations among predictors were allowed per standard SEM practice). Ethnic and linguistic α diversities are more strongly related to each other than either of them is to religious diversity, but even the ethnic to linguistic correlation is only around 0.5. For β diversity, ethnic and linguistic diversity are moderately related to each other and essentially unrelated to religious diversity. Thus, treating each kind of diversity separately in the overall model is justified.

**Table 1:** Select standardized total, direct, and indirect effects from the SEM model presented in Figure 2.

Effects	Estimate
*Effects of Linguistic β Diversity on Instability*	
Total	0.635
Direct	0.707
Indirect (all paths combined)	−0.072
*Interactive Effects of Religous β Diversity on Instability*	
Total	−0.497
Direct	−0.497
Indirect (all paths combined)	0.0
*Effects of Linguistic α Diversity on Instability*	
Total	−0.200
Direct	0.0
Indirect (all paths combined)	−0.200
*Effects of Ethnic α Diversity on Instability*	
Total	0.237
Direct	0.197
Indirect (all paths combined)	0.040
*Effects of GDP on Instability*	
Total	−0.473
Direct	−0.376
Indirect (through Gini)	−0.097

Notable significant pathways in Figure 2 are the following: Linguistic α diversity has a negative effect on GDP, which in turn has a negative effect, both directly and via economic inequality (GINI), on societal instability. We found no evidence to support the possibility that instability actually drives either GDP or GINI. There is also a weak but significant effect of ethnic α diversity on societal instability that is not mediated by economic conditions, in the form of the ethnic Simpson index to Societal Instability pathway.

**Figure 3:**
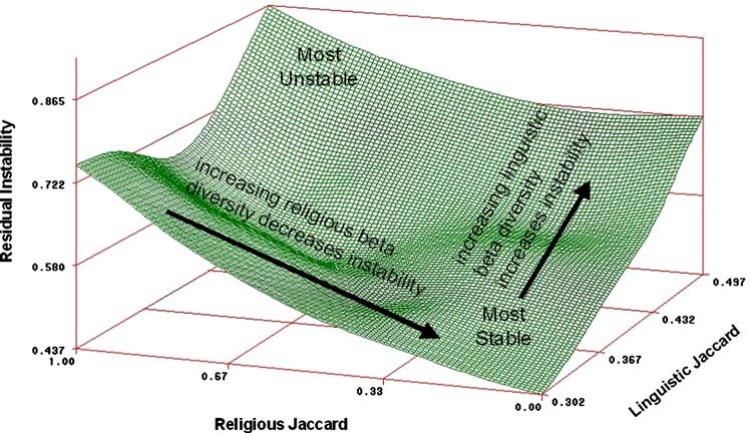
Visualization of interactive effects of linguistic β diversity and religious β diversity on societal instability.

For β diversity, there is a substantial positive effect of linguistic diversity on instability in the model. Thus, nations whose linguistic composition is very different from that of their neighbors tend to have more internal strife. However, there is an interaction with religious diversity, which has an effect in the opposite direction. Increasing religious β diversity is associated with decreased societal instability. In other words, the more religiously different a nation is from its neighbors, the more internally stable it is. As a result of this interaction, the most stable nations are those that are religiously unique, but linguistically similar to their neighbors. This interaction is represented in Figure 3.

**Figure 4:**
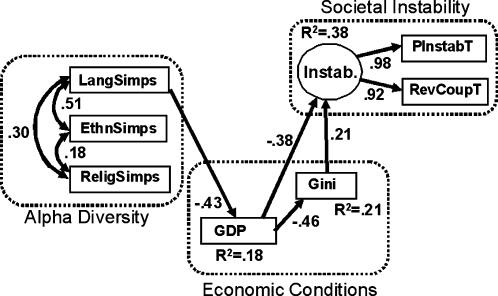
SEM results for model including only α diversity measures (for comparison against Fig. 2).

We reran our analyses removing the β diversity variables, in order to make a more direct comparison with previous studies, which considered only α diversity measures (Figure 4). With β measures absent, α pathway coefficients differ. Notably, the only significant effects of cultural diversity on societal instability are now the indirect ones via economic conditions, whereas in the full model there is also a weak direct effect. Additionally, the inclusion of β diversity in the model substantially increases the variance explained in societal instability from 38% to 58%.

## Discussion

The key findings of our structural equation models are the following. First, there is an effect of within-country diversity on societal instability, with more diversity being associated with more instability. This is a novel result, as previous studies have taken economic performance as their outcome variables, rather than examining societal instability as we do here.

The effect of within-country diversity on societal instability is largely mediated (and moderated) by economic conditions. We observe a negative relationship between within-country diversity and economic performance. This aspect of our findings replicates previous studies [1], [5], [7], and we also show that poor economic performance and high economic inequality are in turn associated with societal instability. Why ethno-linguistic diversity in particular should be correlated with poor economic performance is a question economists continue to investigate [2], [3]. Our results confirm previous findings that the best mechanisms for reducing social and political instability will be those that increase economic growth, because of the strong direct link from GDP to social instability.

Second, our results indicate that the different types of diversity are not perfectly correlated and have different effects. Ethnic and linguistic diversities are moderately correlated, and it is linguistic rather than ethnic diversities that have the strongest relationships to other variables. We suspect that this may be due to the superior quality of the linguistic data, since languages are better-studied and easier to define than ethnic groups. Religious α diversity does not have the same negative effects on economic performance as linguistic α diversity. Alesina and coworkers [7] report similar results and suggest that multiple religions are a hallmark of pluralistic and relatively developed nations.

Our most novel finding concerns the previously-unstudied effects of β diversity on societal instability. The more linguistically different from its neighbours, the more unstable a nation becomes, whilst religious β diversity produces an opposing effect. The more religiously distinct a nation is from its neighbours, the less instability is created by linguistic diversity. We note that these effects, unlike the effects of α diversity on societal instability, are not mediated by economic performance. We therefore suspect they operate at a more cultural or psychological level.

We are curious why religious, but not linguistic, differentiation from neighbours should produce a stabilizing effect. Alexander [19] argued that religions are cultural inventions which function to extend nations, suggesting that the unit of a ‘nation’ is an emergent property of unifying and distinctive belief systems. It therefore may be that a shared religious or moral system within a country which differs from those of surrounding countries leads to a sense of shared identity, common purpose and harmony. Such effects could work on multiple scales, from the sub-group within a city that belongs to a particular church, to larger-scale denominational differences, right up to differences between faiths across national boundaries. Support for this explanation must come from further analyses of within- and between-nation diversity.

There are some limitations to the study which should be acknowledged. First, our model shows the direction of the causal influence between economic performance and societal instability as flowing from the former to the latter. This is likely an oversimplification, as a feedback from instability to economic performance would be expected. This result only means that effects of economics on instability are predominant, not exclusive. Finer-grained data, and in particular time series, would allow more detailed investigation of the causal nexus between economics and societal strife.

A second limitation is that we do not consider the role of the institutional environment. Previous research suggests that national institutional quality can have a major impact on outcomes, and in particular moderates the relationship between within-country diversity and economic performance [2]. Both data availability and conceptual simplicity–basically, the difficulty of specifying where institutional quality should fit in Figure 1 and what its causal relationships to other variables should be (see [7])-have prevented us from including institutional measures here, but this should be a priority for further investigation. These limitations, noted, we hope the relationships observed here will be useful in stimulating further comparative and historical work on the building and functioning of nations.
